# Effects of prolonged local vibration superimposed to muscle contraction on motoneuronal and cortical excitability

**DOI:** 10.3389/fphys.2023.1106387

**Published:** 2023-01-12

**Authors:** Clara Pfenninger, Nathan Grosboillot, Guillaume Digonet, Thomas Lapole

**Affiliations:** ^1^ Laboratoire Interuniversitaire de Biologie de la Motricité, Lyon 1, Université Savoie Mont-Blanc, Université Jean Monnet Saint-Etienne, Saint-Étienne, France; ^2^ HAVAE EA6310, Faculty of Science and Technology, University of Limoges, Limoges, France; ^3^ University Lyon, UCBL-Lyon 1, Laboratoire Interuniversitaire de Biologie de la Motricité, EA 7424, Université Claude Bernard Lyon 1, Villeurbanne, France

**Keywords:** local vibration, motoneuron excitability, cortical excitability, flexor carpi radialis, transcranial magnetic stimulation

## Abstract

**Introduction:** Acute effects of prolonged local vibration (LV) at the central nervous system level have been well investigated demonstrating an altered motoneuronal excitability with a concomitant increase in cortical excitability. While applying LV during isometric voluntary contraction is thought to optimize the effects of LV, this has never been addressed considering the acute changes in central nervous system excitability.

**Materials and Methods:** In the present study, nineteen healthy participants were engaged in four randomized sessions. LV was applied for 30 min to the relaxed flexor carpi radialis muscle (VIB_RELAXED_) or during wrist flexions (i.e. intermittent contractions at 10% of the maximal voluntary contraction: 15 s ON and 15 s OFF; VIB_CONTRACT_). A control condition and a condition where participants only performed repeated low-contractions at 10% maximal force (CONTRACT) were also performed. For each condition, motor evoked potentials (MEPs) elicited by transcranial magnetic stimulation and cervicomedullary evoked potentials (CMEPs) elicited by corticospinal tract electrical stimulation were measured before (PRE) and immediately after prolonged LV (POST) to investigate motoneuronal and corticospinal excitability, respectively. We further calculated the MEP/CMEP ratio as a proxy of cortical excitability.

**Results:** No changes were observed in the control nor CONTRACT condition. At POST, CMEP decreased similarly in VIB_RELAXED_ (−32% ± 42%, *p* < .001) and VIB_CONTRACT_ (−41% ± 32%, *p* < .001). MEP/CMEP increased by 110% ± 140% (*p* = .01) for VIB_RELAXED_ and by 120% ± 208% (*p* = .02) for VIB_CONTRACT_ without differences between those conditions.

**Discussion:** Our results suggest that LV to the flexor carpi radialis muscle, either relaxed or contracted, acutely decreases motoneuronal excitability and induces some priming of cortical excitability.

## Introduction

In recent years, the use of prolonged (i.e., from a few min to 60 min) local vibration (LV) has emerged as a new rehabilitation method (Alashram et al., 2019; Wang et al., 2020; Avvantaggiato et al., 2021). LV applied to a muscle or its tendon produces repetitive changes in muscle length, inducing in turn muscle spindle Ia afferents discharge (Burke et al., 1976a) that project at both spinal and cortical levels, with the potential for both acute and chronic neuromuscular adaptations (Souron et al., 2017a). While most of the studies on effects of LV have been obtained while applying LV to relaxed muscles (Noma et al., 2012; Tavernese et al., 2013), there exist alternative studies within the literature that applied LV on contracted muscles (Marconi et al., 2011; Celletti et al., 2017), considering that contraction may increase Ia afferents discharge (and likely optimize LV effects) through increased α-γ co-activation (Burke et al., 1976b). In a recent systematic review including 12 studies investigating the potential for LV to induce some motor conditioning, it was accordingly suggested superimposing LV to muscle contraction may be more effective (Fattorini et al., 2021). Yet, there is a paucity of studies comparing LV applied on relaxed vs. contracted muscles (Filippi et al., 2009; Brunetti et al., 2012) so that cumulative evidence is needed. One way to question the potential benefit of superimposing LV to contraction is to investigate the well-known LV-induced acute changes in central nervous system excitability.

For instance, a decrease in spinal loop excitability (i.e., depression in Hoffmann reflex amplitude) has been largely reported after prolonged LV for lower limb muscles, i.e., mainly the soleus muscle (Ekblom and Thorstensson, 2011; Souron et al., 2019), but also for upper limb muscles, i.e., flexor carpi radialis (FCR) muscle (Nito et al., 2021), even when LV was applied during contraction (Rocchi et al., 2018). H-reflex is commonly used as a proxy of motoneuronal excitability but it can be influenced by different mechanisms as presynaptic inhibition or homosynaptic post-activation depression, limiting its interpretation (Misiaszek, 2003; Knikou, 2008).

In recent studies we used corticospinal tract electrical stimulation to directly assess motoneuron excitability after prolonged LV applied either to the muscle belly or its tendon (Souron et al., 2017b; Souron et al., 2019; Kennouche et al., 2021). This is possible because stimulation of the descending tracts directly activates large diameter axons which are not subjected to presynaptic inhibition (Taylor, 2006). After prolonged LV, we suggested a decrease in motoneuronal excitability as evidenced by a decrease in amplitude of evoked responses to corticospinal tract electrical stimulation in the quadriceps (Souron et al., 2017b; Kennouche et al., 2021) and soleus (Souron et al., 2019) muscles. This was confirmed for the FCR muscle, with evoked responses to cervico-medullary electrical stimulation (i.e., cervico-medullary evoked potentials, CMEPs) being decreased after 6 and 20 min of LV (Nito et al., 2021), with no study investigating the influence of contraction on the modulation of motoneuronal excitability after prolonged LV.

Besides changes in motoneuronal excitability, LV may also alter corticospinal excitability which can be investigated through recording of motor evoked potentials (MEPs) evoked by transcranial magnetic stimulation (TMS). For instance, TMS is a well-known non-invasive method to investigate use-dependent plasticity in corticospinal excitability following a variety of interventions (Moscatelli et al., 2021). Yet, previous studies did not show any MEP modulation immediately after prolonged LV to the Achilles tendon (Lapole et al., 2012) or to the FCR (Steyvers et al., 2004) despite greater MEP amplitude 30–60 min after the end of LV exposure. Conversely, it was reported an increase in MEP amplitude immediately after 10 min of LV superimposed to finger contraction and this was still persistent 30 min after vibration cessation (Christova et al., 2010), yet it was conversely reported a decrease in MEP amplitude 30 min after a LV exposure superimposed to wrist flexion (Marconi et al., 2008). Because MEP amplitude depends on the whole corticospinal pathway (Devanne et al., 1997), results regarding corticospinal excitability changes after LV are difficult to interpret because of confounding effects prolonged LV could have at both supraspinal (i.e. cortical excitability) and spinal (i.e. motoneuronal excitability) levels (Souron et al., 2017a). Comparing changes in CMEP and MEP responses may allow to distinguish neural changes origin between supraspinal and motoneuronal levels, the MEP/CMEP ratio being a proxy of cortical excitability (i.e., an increase in the MEP/CMEP ratio would suggest an increase in cortical excitability) (Taylor et al., 2002; McNeil et al., 2013; Pearcey et al., 2014; Brownstein et al., 2021). Using such ratio, we previously suggested increased cortical excitability after prolonged LV to the quadriceps muscle (Souron et al., 2017b; Kennouche et al., 2021).

Altogether, while greater LV effects could be hypothesised to occur when combined to contraction (i.e. due to increased Ia afferent discharge), studies from the literature do not allow to clearly determine whether applying LV on relaxed or contracted muscles would lead to similar acute adaptations within the central nervous system. The aim of our study was therefore to investigate the acute changes of FCR motoneuronal and cortical excitability following prolonged LV superimposed or not to voluntary wrist flexion. To this end, we recorded CMEP and MEP responses and we further calculated MEP/CMEP ratios. We hypothesized that LV would decrease CMEP and increase MEP/CMEP, with superimposed LV to wrist flexion leading to greater effects.

## Materials and methods

### Participants

Nineteen healthy participants (11 men and 8 women; age: 27.7 ± 6.7 years; stature: 171.3 ± 10.3 cm; mass: 67.7 ± 12.1 kg; 2 left-handed) were included in the experiment. All participants were free from neurological disease and musculoskeletal injury, and had no contraindications to TMS (Rossi 2011). The study was approved by the institutional ethics committee (CPP SudEst I; 1408208) and was conformed to the *Declaration of Helsinki*, written informed consent was obtained from each participant prior to the study begin.

### Experimental design

**FIGURE 1:**
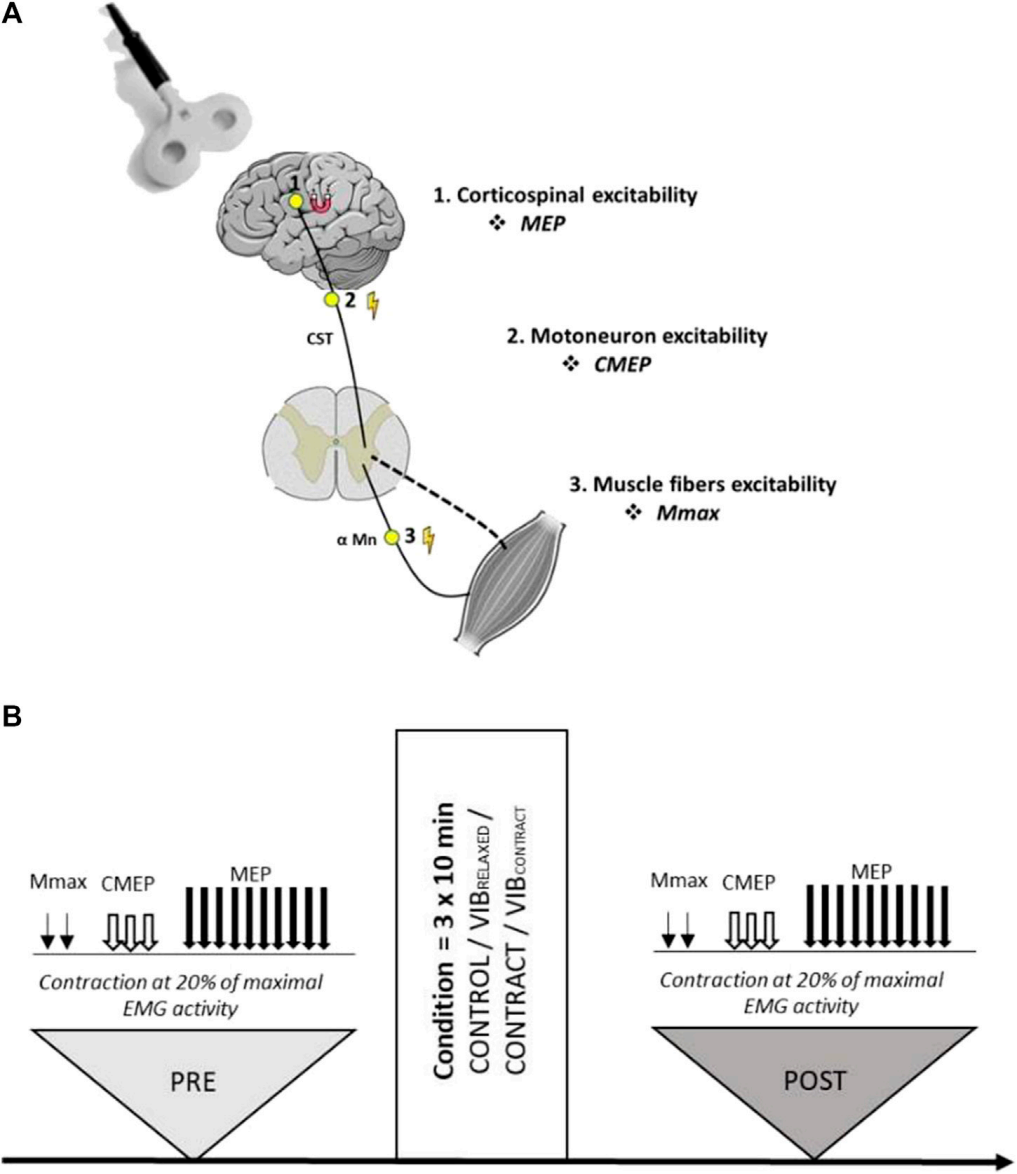
**(A)** Sites of stimulations for each evoked response. **(B)** Overview of the experimental design. Two Mmax, three CMEPs and ten MEPs were evoked during a low-level contraction of the flexor carpi radialis muscle. All measurements were performed with the same order before (PRE) and after (POST) the control, vibration (VIB_RELAXED_), contraction (CONTRACT) or vibration + contraction (VIB_CONTRACT_) conditions.

Participants visited the laboratory on five separate sessions, including a familiarization session followed by the four experimental sessions randomly ordered (CONTROL, vibration on relaxed muscle (VIB_RELAXED_), contraction (CONTRACT) and vibration during muscle contraction (VIB_CONTRACT_) sessions, see below for more details). Sessions were performed at the same time of the day with two to 7 days between sessions. During the familiarization visit, participants were familiarized with all the neuromuscular assessment procedures. As illustrated in Figure 1, experimental sessions comprised neuromuscular assessments before (PRE) and after (POST) each condition. Measurements included the recordings of motor evoked potentials (MEPs; measure of corticospinal excitability), cervico-medullary evoked potentials (CMEPs; measure of motoneuronal excitability) and M-wave (measure of muscle fibers excitability) (Figure 1A). All the measurements were performed on the right upper limb during a low-intensity contraction corresponding to 20% of maximal electromyographic (EMG) activity.

### Experimental procedures

The baseline measurements (PRE) began with a standardized warm up of ten submaximal isometric contractions, gradually increasing the force produced to approach the maximum wrist flexion force. Then, participants performed three 5-s MVCs separated by 60 s of rest. During MVCs, participants were instructed to contract as hard as possible and were verbally encouraged by the experimenter. During MVCs, we recorded the FCR EMG signal. Maximal EMG activity was defined as the mean root mean square value calculated over 200 ms around the force plateau of the best MVC. The target for the on-going constant EMG contraction during which stimulations were delivered was set at 20% of this maximal EMG activity. The contraction was performed at a constant level of EMG activity rather than at a constant force level with the major objective of keeping muscle activation as stable as possible (Brownstein et al., 2021). The root mean square of the EMG activity was displayed on a screen with a guideline set at 20% of the maximal EMG activity.

First, FCR maximal M-wave (Mmax) was measured during the on-going contraction before optimal stimulation intensities for evoking CMEP and MEP responses were determined. Intensities were set at PRE to match approximately 15% of Mmax, with the objective to test the same proportion of the motoneuron pool within and between subjects. Once all appropriate intensities had been determined, baseline measurements were performed. This consisted of two Mmax, three CMEPs, and ten MEP evoked during the low-level contraction of 20% of the maximal EMG activity previously defined (Figure 1B). Each evoked potential was performed during a single contraction, lasting approximatively 3 s, with 10s of rest between trials. The order of stimulations (i.e., MEP, CMEP, M-wave) was always the same (i.e., no randomization). The same measurements, keeping the intensity of stimulation defined at PRE, were performed at POST. At each timepoint, experimental procedures lasted approximately 5–7 min in total.

## Conditions

**FIGURE 2:**
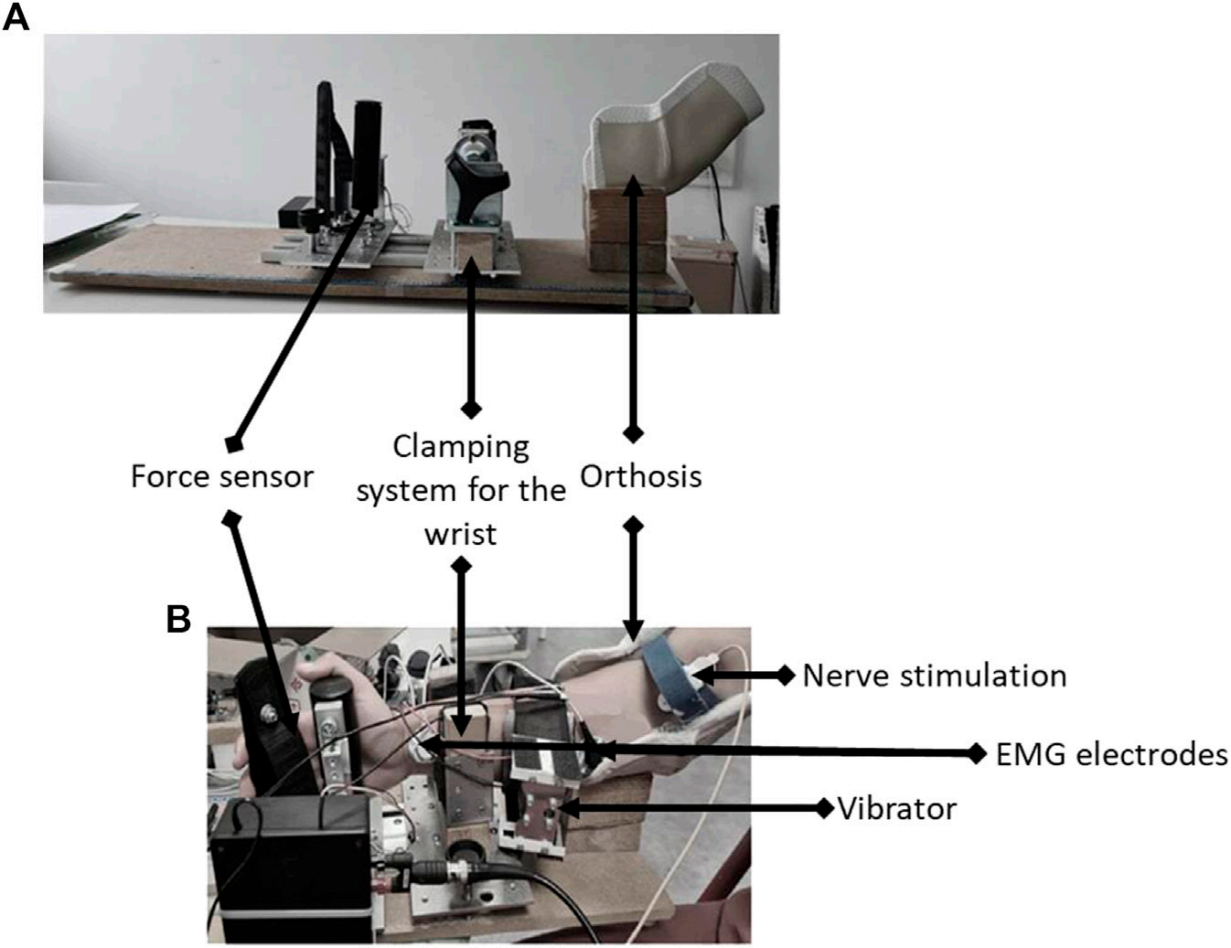
Illustration of the custom-built ergometer **(A)** with placement of the vibrator and nerve bipolar bar stimulating electrode **(B)**.

For the vibration condition (VIB_RELAXED_), LV at a fixed frequency of 100 Hz (VCAR0044-0075-00, SUPT Motion) was applied to the muscle belly of the relaxed right FCR (Figure 2). The application lasted 10 min and was repeated three times with an interval of 1 min as described in previous studies (Marconi et al., 2008; Tavernese et al., 2013; Celletti et al., 2017).

For the contraction condition (CONTRACT), participants were asked to voluntarily perform isometric wrist flexion at 10% MVC (thanks to a visual feedback of the force trace). Participants had to contract for 15 s, then relax for 15 s, and this was repeated for 10 min three times with an interval of 1 min in-between. The “contract/relax” rhythm was provided to the participant by an audio soundtrack.

For the vibration + contraction condition (VIB_CONTRACT_)**,** a combination of LV and muscle contraction was performed. Participants had to contract and relax as presented above for 10 min three times and LV was applied continuously on the right FCR during the 10 min periods.

For the control condition (CONTROL), participants arm was installed in the ergometer with the right FCR fully relaxed for 30 min.

## Instrumentation

### Torque recordings

Voluntary isometric wrist flexion force was recorded with a custom-built ergometer. Participants were comfortably seated in a chair in front of an adjustable table where the ergometer was placed (Figure 2). Right arm of participants was blocked in an orthosis with an elbow angle of 120°, a shoulder abduction of 20°, and no shoulder flexion. The forearm was locked in a semi-pronation position with a clamping system at the wrist and a force sensor positioned in the palm of the hand to measure the strength in wrist flexion. Positions of the wrist clamp and the force sensor were marked on a meter to ensure reproducibility between sessions. This position was maintained throughout the entire session.

### Electromyography (EMG)

Participants were first prepared by shaving, gently abrading the skin, and then cleaning it with isopropyl alcohol. EMG of the FCR was recorded with a pair of self-adhesive surface electrodes (Meditrace 100, Covidien, Mansfield, MA) in a belly-tendon montage. The reference was placed on the ulna styloid process. Signal was bandpass filtered (10–500 Hz), amplified by bio-amplifier (ML138, ADInstruments; common mode rejection ration = 85 db, gain = 5000) and analogue-to-digitally converted at a sampling rate of 2000 Hz by Powerlab system (16/30-ML880/P, ADInstruments, Bella Vista, Australia). All data were analysed offline using Labchart 8 software (ADInstruments).

### Peripheral nerve stimulation: Mmax

The right median nerve was stimulated by a single rectangular electrical stimulus with a duration of 0.2 ms (Brownstein et al., 2020) and a maximum output voltage of 400 V (DS7A, Digitimer, Welwyn Garden City, Hertfordshire, United Kingdom) delivered through a bipolar bar stimulating electrode with 30 mm anode–cathode spacing (Bipolar Felt Pad Stimulating Electrode Part Number E. SB020/4 mm, Digitimer) placed at the cubital fossa (Figure 2B).

Electrical stimuli were first administered at 5 mA and then were increased by 5-mA steps until the maximal M-wave amplitude (Mmax) was obtained on the right FCR during the 20% maximal EMG activity contraction previously defined. The optimal intensity was then increased by 20% to ensure supramaximal stimulation.

### Cervico-medullary electrical stimulation: CMEP responses

The single rectangular electrical stimulus of 0.2 ms duration (DS7A, Digitimer, Welwyn Garden City, Hertfordshire, United Kingdom) was delivered through electrodes positioned near the mastoid processes with the anode on the right side to activate axons at the cervicomedullary junction (Brownstein et al., 2020)*.* The stimulation intensity was adjusted to elicit a CMEP amplitude of ∼15% of Mmax during the 20% maximal EMG activity contraction previously defined.

### Transcranial magnetic stimulation: MEP responses

The left motor cortex was stimulated by a figure-of-eight coil connected to a Magstim 200 magnetic stimulator (Magstim Co., Ltd., Whitland, United Kingdom). The coil was positioned tangentially to the scalp (at a 45° angle to the midline) to induce a posterio-anterior current. The optimal position of the coil was determined as the position evoking the greatest MEP in response to a TMS pulse at 50% of maximum. Once identified, this position was marked directly on a swimming pool cap worn by participants to ensure consistent positioning throughout the experiment. Subsequently, the TMS intensity was adjusted to elicit a MEP of ∼15% of Mmax during the 20% maximal EMG activity contraction previously defined.

### Data analysis

At each timepoint (i.e. PRE and POST), the mean peak-to-peak amplitudes of the two Mmax**,** three CMEP and ten MEP responses were used for statistical analysis. CMEP and MEP amplitudes were expressed in percentage of their corresponding Mmax amplitude and were considered as a proxy of motoneuronal and corticospinal excitability, respectively. To quantify changes in cortical excitability, MEP/CMEP ratios were further calculated (Pearcey et al., 2014; Brownstein et al., 2021; Kennouche et al., 2021).

### Statistical analysis

All data are presented as mean values ±SD. CMEP, MEP and MEP/CMEP ratios were log transformed for statistical analysis. Normal distribution and homogeneity of variances were checked on transformed data using Shapiro–Wilk and Levene tests, respectively.

Then two-way repeated-measures ANOVAs were used [time (PRE, POST) x condition (CONTROL, VIB_RELAXED_, VIB_CONTRACT_, CONTRACT)] to compare each evoked response between conditions. Sphericity was tested using the Mauchly test, and if violated, the correction of Greenhouse-Gasser was applied. In case of significant effect found by the ANOVA, a post-hoc of TukeyHSD with correction for multiple comparisons was carried out. Partial eta squared (η^2^_p_) was calculated to estimate effect sizes, with values representing small (η^2^_p_ ≥ 0.1), medium (η^2^_p_ ≥ 0.25), and large (η^2^_p_ ≥ 0.40) effects.

For all statistical analyses, the level of significance was set at *p* < 0.05. Statistical analysis was performed using R software (version 1.3.1093).

## Results

### CMEP responses

**FIGURE 3:**
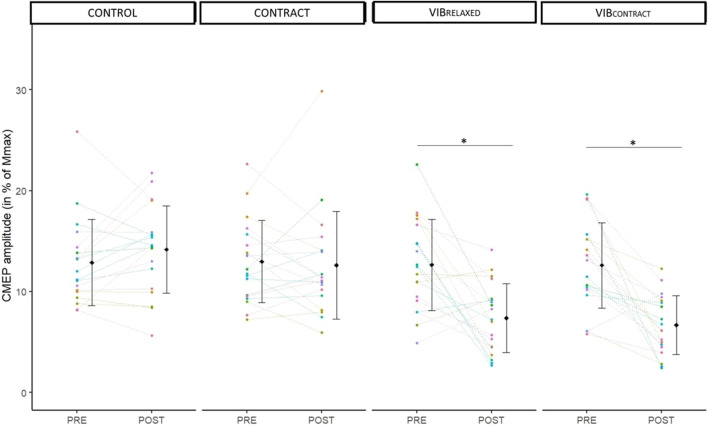
Mean and individual CMEP amplitudes in % of Mmax. PRE and POST values are represented for each condition. Each dot represents the value of a participant. * represents significant differences between PRE and POST.

Two-way repeated measures ANOVA showed a significant effect of time (F_1,18_ = 29.46, *p* < .001, *η*_p_^2^ = .62), condition (F_3,54_ = 11.14, *p* < .001, *η*_p_^2^ = 0.38) and time x condition (F_1.9,34.4_ = 11.78, *p* < .001, *η*_p_^2^ = .39). A significant CMEP depression was found from PRE to POST for VIB_RELAXED_ (−32% ± 42%, *p* < .001) and VIB_CONTRACT_ (−41% ± 32%, *p* < .001) without differences for CONTROL (+13% ± 29%, *p* = .99) and CONTRACT (-1% ± 29%, *p* = .99) (Figure 3 **)**. POST values of VIB_RELAXED_ and VIB_CONTRACT_ were not different (*p* = .99).

### MEP responses

**FIGURE 4:**
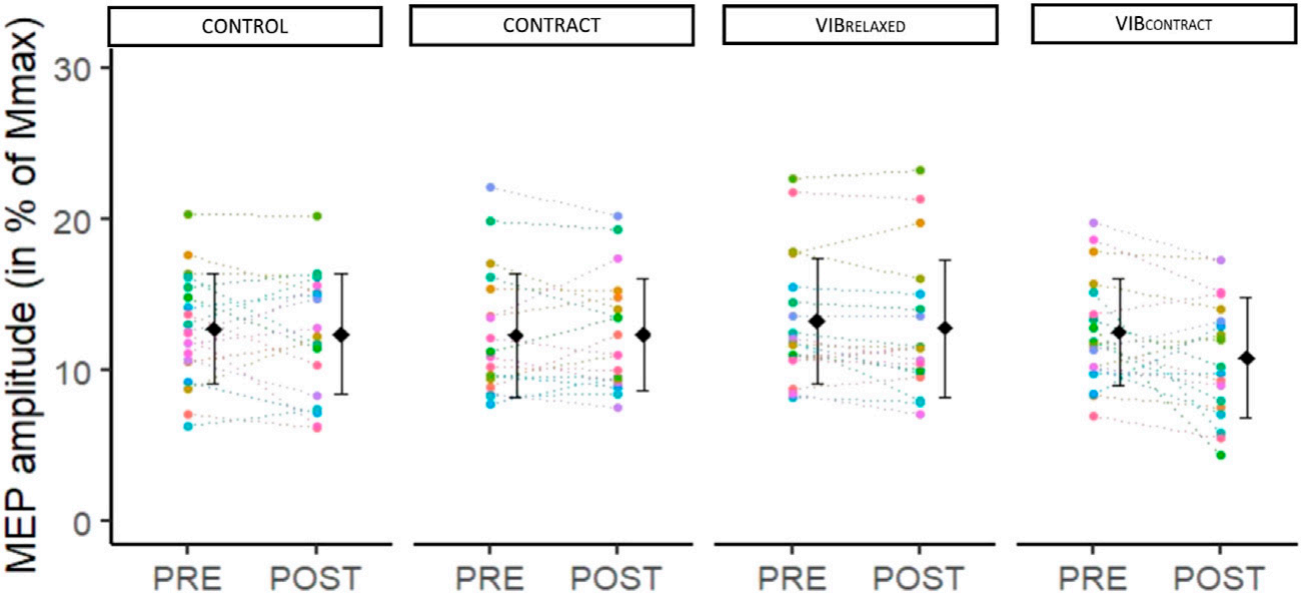
Mean and individual MEP amplitudes in % of Mmax. PRE, POST and POST30 values are represented for each condition. Each dot represents the value of a participant.

Two-way repeated measures ANOVA did not show any significant effect of condition (F_3,54_ = .64, *p* < .60, *η*_p_^2^ = .04). Although a significant main effect of time was found (F_1,18_ = 4.74, *p* < 0.04, *η*_p_^2^ = .21), there were no differences between PRE and POST (*p* = .2). Moreover, time × condition interaction was not significant (F_2.08,37.47_ = 2.21, *p* = 0.12, *η*_p_^2^ = .11) (Figure 4 **).**

### MEP/CMEP ratio

**FIGURE 5:**
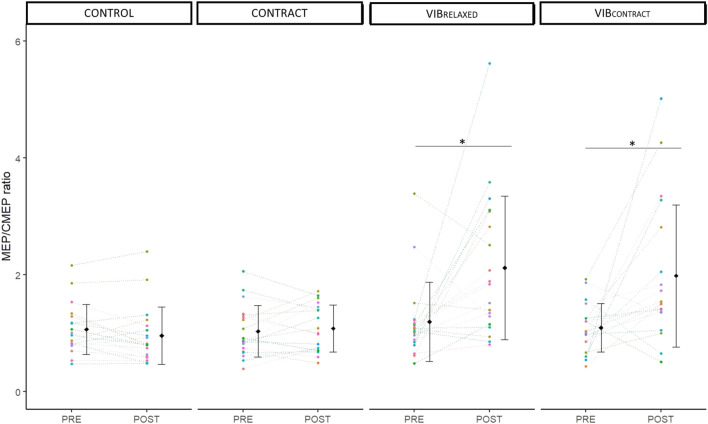
Mean and individual MEP/CMEP ratio. PRE and POST values are represented for each condition. Each dot represents the value of a participant. * represents significant differences between PRE and POST.

Two-way repeated measures ANOVA showed a significant effect of time (F_1,18_ = 14.10, *p* < .001, *η*_p_^2^ = .44), condition (F_3,54_ = 9.65, *p* <.001, *η*_p_^2^ = .35) and time x condition (F_2.1,37.79_ = 6.82, *p* < 0.01, *η*_p_^2^ = .28). Post-hoc testing showed no differences at baseline between conditions (*p* = 1). A significant MEP/CMEP increase was found from PRE to POST for VIB_RELAXED_ (+110 ± 140%, *p* = .01) and VIB_CONTRACT_ (+120 ± 208%, *p* = .02) without differences for CONTROL (−9 ± 24%, *p* = .98) nor CONTRACT (10 ± 37%, *p* = .99) (Figure 5 **)**. POST values of VIB_RELAXED_ and VIB_CONTRACT_ were not different (*p* = .99)**.**

## Discussion

The aim of the present study was to investigate the influence of 30 min of LV superimposed or not to voluntary wrist flexion on acute changes in motoneuronal and corticospinal excitability. To this purpose, we recorded CMEP and MEP responses of similar size during a low-intensity contraction and we further calculated MEP/CMEP ratios. Immediately after LV applied both during a relaxed state and during wrist flexion, we observed a decrease in CMEP responses, suggesting a decrease in motoneuronal excitability, with no changes after wrist flexion only. MEP amplitude were not modulated suggesting a greater cortical excitability after the two LV conditions (i.e., an increase in MEP/CMEP ratio).

From a methodological point of view, all responses recorded in the present study were evoked during a low-intensity contraction by considering a constant level of electromyographic activity. This allowed us to maintain a stable muscle activation across timepoints (Brownstein et al., 2021). Similarly, we adjusted stimulations intensities at baseline trying to target the same peak-to-peak amplitude for all the evoked responses (i.e., CMEP and MEP) (Pearcey et al., 2014; Kennouche et al., 2021). This contrasts with one of our previous studies where the size of the responses to TMS and corticospinal tract electrical stimulation were not matched, leading us to likely test different proportions of the motoneuron pool (Souron et al., 2017b). We acknowledge that we were not able in the present study to systematically match the size of the evoked responses, as demonstrated by MEP/CMEP ratios far from the expected value of 1 for this ratio at baseline (i.e., PRE) for some participants, likely because of responses variability and difficulties in evoking large CMEPs in some participants.

As expected, CMEPs responses remained similar between PRE and POST measurements in the CONTROL condition, so they were in the CONTRACT condition (i.e., 10% MVC). This latter result is in agreement with previous findings of unchanged responses to corticospinal tract stimulation after repeated low-intensity contraction (i.e., 5% MVC) (Petersen et al., 2003). After prolonged FCR LV (i.e., VIB_RELAXED_ condition), we observed a 32% decrease in CMEP amplitude, as also previously reported on upper limb (Nito et al., 2021) and lower limb (Souron et al., 2017b; Souron et al., 2019; Kennouche et al., 2021) muscles, suggesting decreased motoneuronal excitability as a consequence of repeated Ia afferents inputs onto to alpha-motoneurons during LV (Souron et al., 2017b; Kennouche et al., 2021; Nito et al., 2021). While we initially hypothesized greater CMEP depression in the VIB_CONTRACT_ than VIB_RELAXED_ condition, the 41% decrease in CMEP amplitude in VIB_CONTRACT_ was not different than the decrease observed in VIB_RELAXED_.

Despite a decrease in CMEPs amplitude after LV, we reported no immediate vibration-induced changes in MEPs amplitude regardless of muscle state. As corticospinal excitability depends on both cortical and spinal levels, MEP amplitude should be interpreted in light of changes at the spinal level (McNeil et al., 2013). In our study, the increase in MEP/CMEP ratios after LV applied on relaxed and contracted muscles suggests an increase in cortical excitability. For instance, MEP responses were not altered after prolonged LV despite the decrease in motoneuronal excitability (i.e., decreased CMEP responses), likely masking an increase in cortical excitability (i.e., increased MEP/CMEP ratios). Similar findings were previously reported by Kennouche et *al.* (2022) and Souron et *al.* (2017) on the quadriceps muscle. One first potential hypothesis to explain the observed LV-induced increased cortical excitability could be an increase in descending drive during the low-intensity contraction. For instance, this could have been necessary to reach the target EMG activity despite the LV-induced decrease in motoneuronal excitability. However, this would not explain previous findings of increased resting corticospinal excitability 30–60 min after prolonged LV (Steyvers et al., 2004; Lapole et al., 2012). Therefore, an alternative hypothesis to explain previous findings as well as present results would be cortical excitability priming (i.e., cortical excitability modulation) after prolonged LV, likely through cortical projections of Ia afferents (Mima et al., 1997). This may rely on topographically and functionally specific reciprocal connections between primary somatosensory cortex and primary motor cortex, as suggested in a TMS study by increased excitability of the sensorimotor pathways for some responders to LV (Lapole and Tindel, 2015).

Because a greater discharge of Ia afferents is suggested to occur during LV superimposed to muscle contraction (i.e., VIB_CONTRACT_) than LV applied on relaxed muscle (i.e., VIB_RELAXED_) (Burke et al., 1976b), we initially hypothesized greater CMEP decrease and MEP/CMEP ratio increase after VIB_CONTRACT_ than VIB_RELAXED_. Yet, changes were not different between the two muscle states. This would agree with previous findings where a single session of peripheral nerve electrical stimulation increased MEP amplitude when applied at rest but not when superimposed to isometric voluntary contraction, likely as a result of gating of sensory inputs during isometric contraction (Saito et al., 2014). For instance, gating of sensory inputs is known to occur during voluntary contraction (Rushton et al., 1981; Kakigi et al., 1995), through inhibitory circuits located at both spinal and supra-spinal levels (Seki and Fetz, 2012; Lei et al., 2018). We may therefore speculate that the expected greater Ia afferent discharge in VIB_RELAXED_ did not lead to greater effects because of similar gating of afferent inputs.

Alternatively, the lack of differences between VIB_CONTRACT_ and VIB_RELAXED_ could be the result of an inability of VIB_CONTRACT_ to actually increase Ia afferents discharge when compared to VIB_RELAXED_. In the original study of Burke et *al.* (1976) using microneurography, it was actually demonstrated increased Ia afferents discharge during LV, and this LV-induced increased discharge was even greater when LV was superimposed to an isometric voluntary contraction. Yet, the latter result was only observed for Ia afferents that were responding submaximally during the relaxed state. For those Ia afferents that were already responding one-to-one to LV, voluntary contraction usually had no influence on discharge rate (Burke et al., 1976b). Because the LV characteristics we used in the present study are thought to be already optimal for Ia afferent stimulation (Vedel and Roll, 1982), this may explain why prolonged LV led to similar acute changes in motoneuronal and cortical excitability whether it was applied on the relaxed or contracted FCR.

Yet, when considering studies that investigated the long-term effects of three consecutive days of 30-min LV to the quadriceps in elderly women (Filippi et al., 2009) and female volleyball players (Brunetti et al., 2012), increased leg power (Filippi et al., 2009; Brunetti et al., 2012), decreased knee joint laxity (Brunetti et al., 2012), as well as increased vertical jump (Filippi et al., 2009) were reported to be greater when LV was superimposed to voluntary knee extension than when LV was applied alone. It should be however acknowledged that none of the two aforementioned studies included a group performing contraction only. Moreover, LV was applied while the leg was extended so that the quadriceps muscle was in a shortened length. This may have led to reduced responsiveness of muscle spindles to vibration (Burke et al., 1978; Souron et al., 2018), so that performing a contraction actually increased Ia afferents discharge (Eklund et al., 1964; Eklund and Hagbarth, 1966). Further studies could usefully explore how combining different muscle length and muscle state could influence LV effects.

## Data Availability

The raw data supporting the conclusion of this article will be made available by the authors, without undue reservation.
